# Inhibition of Serum Response Factor Improves Response to Enzalutamide in Prostate Cancer

**DOI:** 10.3390/cancers12123540

**Published:** 2020-11-27

**Authors:** R. William Watson, Haleema Azam, Claudia Aura, Niamh Russell, Janet McCormack, Eva Corey, Colm Morrissey, John Crown, William M Gallagher, Maria Prencipe

**Affiliations:** 1 Conway Institute of Biomolecular and Biomedical Research, UCD School of Medicine, University College Dublin, Belfield, D4, Dublin, Ireland; william.watson@ucd.ie; 2 Cancer Biology and Therapeutics Laboratory, UCD Conway Institute, University College Dublin, Belfield, D4, Dublin, Ireland; haleema.azam@ucdconnect.ie (H.A.); claudia.auragonzalez@ucd.ie (C.A.); niamh.russell@gmail.com (N.R.); William.Gallagher@ucd.ie (W.M.G.); 3 UCD School of Biomolecular and Biomedical Science, University College Dublin, Belfield, D4, Dublin, Ireland; 4 Research Pathology Core, Conway Institute of Biomolecular and Biomedical Research, University College Dublin, Belfield D4, Dublin, Ireland; janet.McCormack@ucd.ie; 5 Department of Urology, University of Washington, Seattle, WA 98195, USA; ecorey@uw.edu (E.C.); cmorriss@uw.edu (C.M.); 6 Department of Medical Oncology, St Vincent’s University Hospital, Dublin, Ireland; john.crown@cancertrials.ie

**Keywords:** enzalutamide, prostate cancer, serum response factor, androgen receptor

## Abstract

**Simple Summary:**

Despite the improvement in the treatment options for prostate cancer patients, we still lack effective treatments when the cancer has spread outside the prostate. Therefore, there is an urgent need to develop new therapies that will work better in this setting. Our study has identified one central factor, the serum response factor (SRF), which controls many genes associated with prostate cancer development. We have shown that an SRF inhibitor can stop the cancer cells from dividing and surviving. When this inhibitor is used in combination with current treatments, these are more effective in killing cancer cells. We also confirmed the relevance of SRF to patients by looking at its abundance in prostate cancer tissues from patients. We showed that patients who did not respond to current treatments had significantly higher levels of SRF. We are currently investigating SRF inhibitor mechanisms and hope that these drugs will soon be available to patients.

**Abstract:**

Castrate-resistant prostate cancer (CRPC) is challenging to treat with the androgen receptor (AR), the main target and key focus of resistance. Understanding the mechanisms of AR interaction with co-regulators will identify new therapeutic targets to overcome AR resistance mechanisms. We previously identified the serum response factor (SRF) as a lead target in an in vitro model of CRPC and showed that SRF expression in tissues of CRPC patients was associated with shorter survival. Here, we tested SRF inhibition in vitro and in vivo to assess SRF as a potential target in CRPC. Inhibition of SRF with the small-molecule inhibitor CCG1423 resulted in enhanced response to enzalutamide in vitro and reduced tumour volume of LuCaP 35CR, a CRPC patient-derived xenograft model. Nuclear localisation of AR post-CCG1423 was significantly decreased and was associated with decreased α-tubulin acetylation in vitro and decreased prostate specific antigen (PSA) levels in vivo. SRF immunoreactivity was tested in metastatic tissues from CRPC patients to investigate its role in enzalutamide response. Kaplan–Meier curves showed that high SRF expression was associated with shorter response to enzalutamide. Our study supports the use of SRF inhibitors to improve response to enzalutamide.

## 1. Introduction

Current treatments for locally advanced and metastatic prostate cancer are mainly focused on targeting the androgen receptor (AR). However, despite initial response, most men progress to develop castrate-resistant prostate cancer (CRPC), which is challenging to treat. Recently, second generation AR blockers, such as abiraterone acetate and enzalutamide, have been introduced into the clinic, but resistance to these treatments also develops [1,2,3,4]. This highlights the need for innovative therapeutic approaches that aim to continue disrupting AR downstream signalling but are not directly targeting the AR itself, thus circumventing AR-related resistance. Among the multiple mechanisms of resistance, aberrations in AR co-regulators represent a promising therapeutic approach since co-regulators are not susceptible to AR resistance mechanisms [5].

Our group and others identified the serum response factor (SRF), a transcription factor involved in cytoskeleton organisation and cellular proliferation [6], as an important factor in prostate cancer progression in vitro and in vivo. We previously showed that inhibition of SRF with siRNA and with the small-molecule inhibitor CCG1423 reduced cell viability of LNCaP Abl cells [7], a cellular model of castrate resistance [8], and that combination with enzalutamide significantly decreased cell viability in comparison with CCG1423 and enzalutamide single treatments [9]. A role for SRF in regulating AR action has been established by Heemers and colleagues, who showed that SRF was involved in regulating AR transcriptional activity through its transcriptional target four-and-a-half LIM domain protein 2 (FHL2) [10]. Moreover, a clinically relevant androgen-dependent gene signature under SRF regulation was identified which could distinguish between indolent and aggressive prostate cancer [11]. Supporting the relationship between SRF and AR, we discovered a negative feedback loop between SRF and AR in the presence of androgens in both LNCaP parental and LNCaP Abl cells [9]. This relationship was confirmed in patients’ tissue samples, with a negative correlation between AR and SRF expression in CRPC bone metastases and a positive correlation in androgen-naïve primary tumours [9].

The relevance of SRF protein expression in prostate cancer patients was demonstrated in several published studies [7,12,13,14], highlighting the clinical relevance of this protein and the possibility of using it as a therapeutic target and a disease biomarker. SRF up-regulation was shown to be an early event during prostate cancer progression, being associated with biochemical recurrence [13], and also a biomarker of progression, with higher levels of SRF associated with castrate resistance [7], decreased survival from diagnosis [12] and response to docetaxel [14].

Due to the potential use of SRF as a therapeutic target in advanced prostate cancer and its relationship with AR, in this study, we tested the combination treatment of enzalutamide and CCG1423 in vitro and in LuCaP 35CR, a patient-derived xenograft (PDX) model of enzalutamide resistance. Similarly to one recognised enzalutamide mechanisms of action [15], inhibition of SRF with CCG1423 resulted in decreased AR nuclear localisation. These data suggest that SRF up-regulation may promote higher AR nuclear localisation and hence higher AR transcriptional activity. In line with this hypothesis, we showed that enzalutamide-resistant patients had higher expression levels of SRF.

## 2. Results

### 2.1. Combined Effect of Enzalutamide and CCG1423 In Vitro

**Figure 1:**
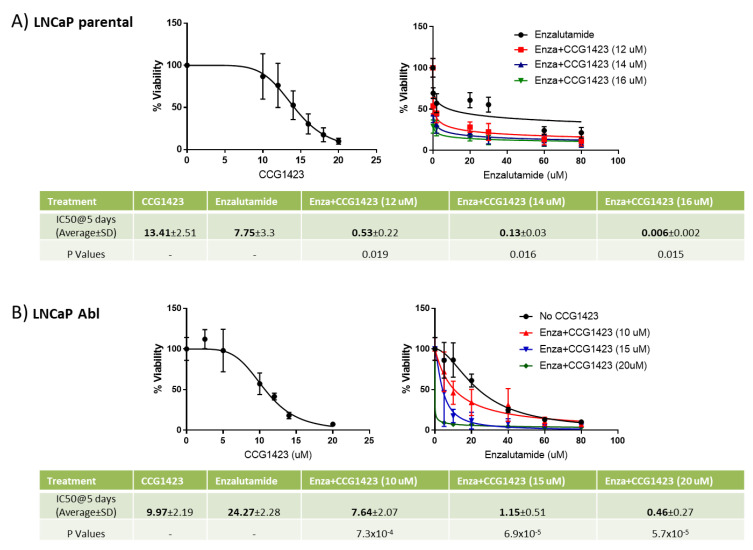
LNCaP cells were seeded in 96 well plates at a density of 3 × 10^3^ cells per well. The following day, they were treated either with vehicle (DMSO), increasing concentrations of CCG1423 alone (parental: 10, 12, 14, 16, 18, 20 μM; Abl: 2.5, 5, 10, 12, 15, 20 μM), increasing concentrations of enzalutamide alone (parental: 0.2, 2, 20, 30, 60, 80 μM; Abl: 5, 10, 20, 40, 60, 80 μM) or all the 36 possible combinations of CCG1423 and enzalutamide. Five days after treatment, cellular viability was measured by 3-(4,5)-dimethylthiazol-2-yl-2,5-diphenyltetrazolium bromide (MTT) assay. IC50 values were calculated using Prism. (**A**) LNCaP parental; (**B**) LNCaP Abl. The graphs show % viability at different concentrations of CCG1423 and enzalutamide from three independent experiments in triplicate. Only three concentrations of CCG1423 are shown in the graph for better visualisation of curves. The tables show average IC50 values from three independent experiments in triplicate ± standard deviation (SD). IC50 values were compared using *t* test assuming equal variance. For each column, *p*-values refer to comparisons between that column and single treatment with enzalutamide.

LNCaP parental and Abl isogenic pair cells were treated with six increasing concentrations of enzalutamide and CCG1423, singly and in combination for 5 days, and cell viability was assessed using 3-(4,5)-dimethylthiazol-2-yl-2,5-diphenyltetrazolium bromide (MTT) assays. This analysis showed that both cell lines are sensitive to CCG1423 with similar half-maximal inhibitory concentration (IC50) values (13.41 ± 2.51 and 9.97 ± 2.19 for LNCaP parental and Abl cells, respectively). However, while LNCaP parental cells show lower IC50 values in response to enzalutamide compared to CCG1423 (7.75 ± 3.3 vs. 13.41 ± 2.51, respectively), the Abl cells, a model for castrate resistance, were more responsive to CCG1423 than enzalutamide with IC50 values of 9.97 ± 2.19 vs. 24.27 ± 2.28, respectively. Moreover, in both cell lines, the addition of CCG1423 to enzalutamide treatment resulted in a significant decrease in enzalutamide IC50 values with increasing concentrations of CCG1423 (Figure 1).

### 2.2. Combined Effect of Enzalutamide and CCG1423 in LuCaP 35CR Patient-Derived Xenograft

**Figure 2:**
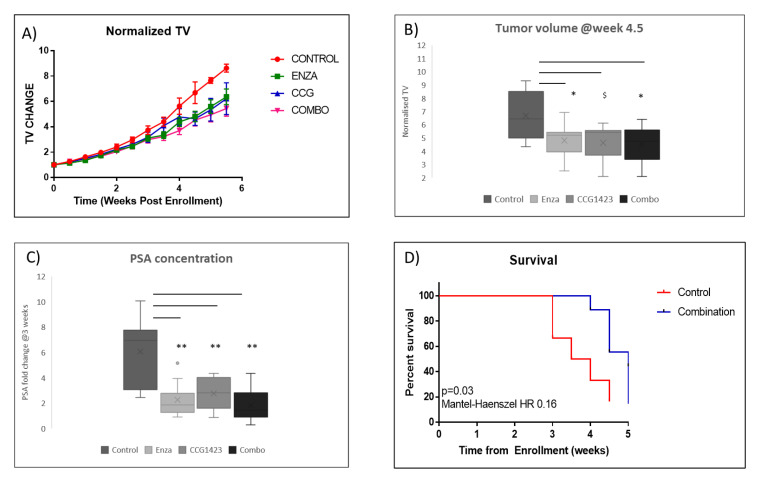
Fox Chase CB17 SCID castrated mice were implanted with LuCaP 35CR tumour fragments. Once tumour volumes exceeded 101 mm^3^, animals were randomised to receive either vehicle control (*n* = 11), enzalutamide (ENZA; 50 mg/kg p.o.; 5 days on, 2 days off; *n* = 11), CCG1423 (CCG; 0.15 mg/kg i.p. 5 days on, 2 days off; *n* = 11) or enzalutamide plus CCG1423 (dosing regimen as previously noted). (**A**): Change in tumour volume with time by treatment group 6 weeks after start of treatment. (**B**): Box plot of tumour volumes by treatment group, 4.5 weeks after start of treatment. (**C**): Box plots of PSA fold change at 3 weeks of treatment. Average PSA values and average tumour volume values at 4.5 weeks for each treatment group were compared using *t* test assuming equal variance. * *p* < 0.05, ** *p* < 0.01, $ *p* = 0.057. (**D**): Kaplan–Meier survival curves from date of enrolment for controls and combination treatment groups (log-rank test, *p* = 0.03, Mantel–Haenszel HR 0.16).

To validate our in vitro data on the efficacy of CCG1423 and its combined effect in reducing cell viability in the LNCaP parental and Abl cells in combination with enzalutamide in vivo, we used the LuCaP 35CR, a prostate PDX model, which shows minimal response to enzalutamide. From week 4.5 following treatments, lower tumour volumes (TV) were observed in the treatment groups (enzalutamide, CCG1423 and combination) in comparison with the control group (Figure 2A). TV at 4.5 weeks were significantly lower following enzalutamide (*p* < 0.05), CCG1423 (*p* = 0.057) and combination (*p* < 0.05) treatments compared with controls (Figure 2B). In line with decreased TV, serum PSA levels at week 3 post-treatments were significantly decreased in enzalutamide (*p* < 0.01), CCG1423 (*p* < 0.01) and combination (*p* < 0.01) groups compared with controls (Figure 2C). While no significant differences in TV or PSA were found between single treatments and the combination, Kaplan–Meier analysis showed that combination treatment provide significant survival benefit compared with the control (*p* = 0.03, Mantel–Haenszel hazard ratio (HR) 0.16) (Figure 2D), while single treatments with enzalutamide or CCG1423 did not improve survival (Figure S1).

### 2.3. Androgen Receptor Nuclear Translocation Is Inhibited by CCG1423 through Inhibition of α-Tubulin Acetylation

**Figure 3:**
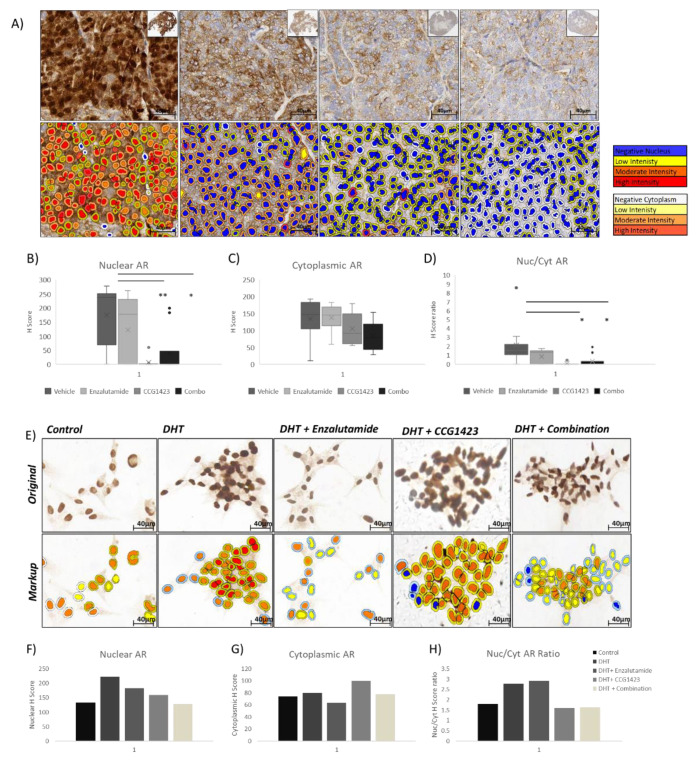
Immunohistochemistry (IHC) staining for androgen receptor (AR) was performed as described in the methods section. (**A**): Representative images of AR staining in the four treatments groups. Top panel shows original images (20× magnification); bottom panel shows the markup images resulting from automatic quantification using Visopharm’s Oncotopix algorithms. (**B**): Nuclear AR protein expression (H score). (**C**): Cytoplasmic AR protein expression (H score). (**D**): Nuclear/cytoplasmic H score ratio. Average AR expression values for each treatment group were compared using one way ANOVA test for cytoplasmic AR staining and Welch’s ANOVA followed by post hoc Games–Howell test for nuclear AR and nuclear/cytoplasmic ratio. ** *p* < 0.01, * *p* < 0.05. (**E**): Immunocytochemistry (ICC) staining for AR in LNCaP Abl cells. Top panel shows original images (20× magnification); bottom panel shows the markup images resulting from automatic quantification using Visopharm’s Oncotopix algorithms. (**F**): Nuclear AR protein expression (H score). (**G**): Cytoplasmic AR protein expression (H score). (**H**): Nuclear/cytoplasmic H score ratio. Images and quantification are representative of three independent experiments. DHT: dihydrotestosterone.

To investigate possible mechanisms for decreased TV and PSA levels following treatments, AR protein expression and sub-cellular localisation in the PDX tumour tissues was investigated by immunohistochemistry (IHC) (Figure 3). This analysis showed that AR nuclear localisation following CCG1423 treatment, singly or in combination with enzalutamide, was significantly decreased (*p* < 0.01 for CCG1423 and *p* < 0.05 for the combination) (Figure 3B), while cytoplasmic levels were decreased but did not reach statistical significance (Figure 3C). Importantly, the nuclear/cytoplasmic ratio of AR expression following CCG1423 treatment, singly or in combination with enzalutamide, was significantly decreased (*p* < 0.05 for both CCG1423 single and combination treatment). The effect of CCG1423 on AR sub-cellular localisation was also confirmed in vitro in the LNCaP Abl cells (cellular model of CRPC). Immunocytochemistry (ICC) experiments showed decreased AR nuclear localisation following CCG1423 treatment, both as a single agent and in combination with enzalutamide (Figure 3E–H). These data indicate that treatment with CCG1423 (singly or in combination with enzalutamide) affects AR nuclear localisation.

**Figure 4:**
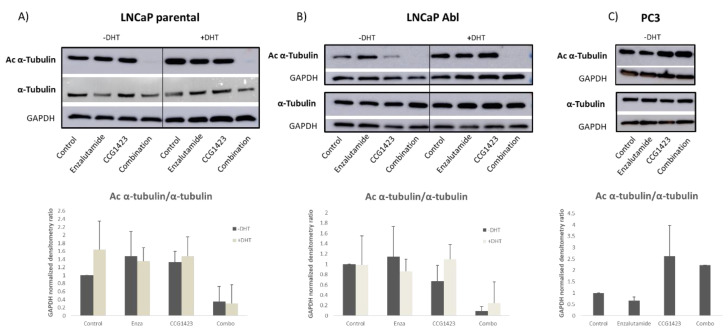
Protein expression of acetylated α-tubulin was assessed by Western Blotting (WB) following treatments as described in the Methods Section. (**A**) LNCaP parental; (**B**) LNCaP Abl; (**C**) PC3. Representative images of acetylated and total α-tubulin expression from three (LNCaP parental and Abl) and two (PC3) independent experiments are shown (top panel). Bottom panels show averages of densitometry analysis of WB images from three (LNCaP parental and Abl cells) and two (PC3) independent experiments ± standard deviation. Bars represent the ratio between acetylated α-tubulin and α-tubulin, following normalisation with GAPDH (to control for loading of proteins).

Next, we looked at possible mechanisms by which SRF inhibition affects AR cellular localisation. AR intracellular localisation can be affected by α-tubulin binding and by levels of acetylated α-tubulin. Increased acetylation of α-tubulin enhances dynein binding to the microtubules [16], which in turn enables AR translocation into the nucleus. αTAT1 is a protein responsible for adding acetyl groups to α-tubulin which is under SRF transcriptional control, with SRF enhancing αTAT1 transcription [17]; therefore, we looked at acetylation levels of α-tubulin in response to treatments as a possible link between SRF inhibition and AR translocation to the nucleus (Figure 4). While only a modest decrease in acetylated α-tubulin was shown in response to CCG1423 as single treatment in LNCaP Abl cells in the absence of dihydrotestosterone (DHT) stimulation (Figure 4B), a significant decrease in α-tubulin acetylation, in response to the combination treatment, was shown in both LNCaP parental (Figure 4A) and Abl (Figure 4B) cell lines in the absence of DHT stimulation (*p* = 0.039 for parental and 0.0003 for Abl). In the presence of DHT, a significant decrease in acetylation of α-tubulin was shown for LNCaP parental (*p* = 0.05) (Figure 4A), while the decrease observed in the Abl cells was not statistically significant (*p* = 0.077) (Figure 4B). To control for the effects of CCG1423 in an AR negative cell line, the level of acetylated α-tubulin in PC3 cells was also assessed (Figure 4C). Since PC3 cells do not express AR, DHT treatment was not included for these cells. Interestingly, in an AR-negative environment, CCG1423 treatment resulted in increased levels of α-tubulin acetylation, in contrast with the decrease observed in LNCaP cells.

### 2.4. SRF Expression Is Associated with Enzalutamide Resistance in Patients with CRPC

**Figure 5:**
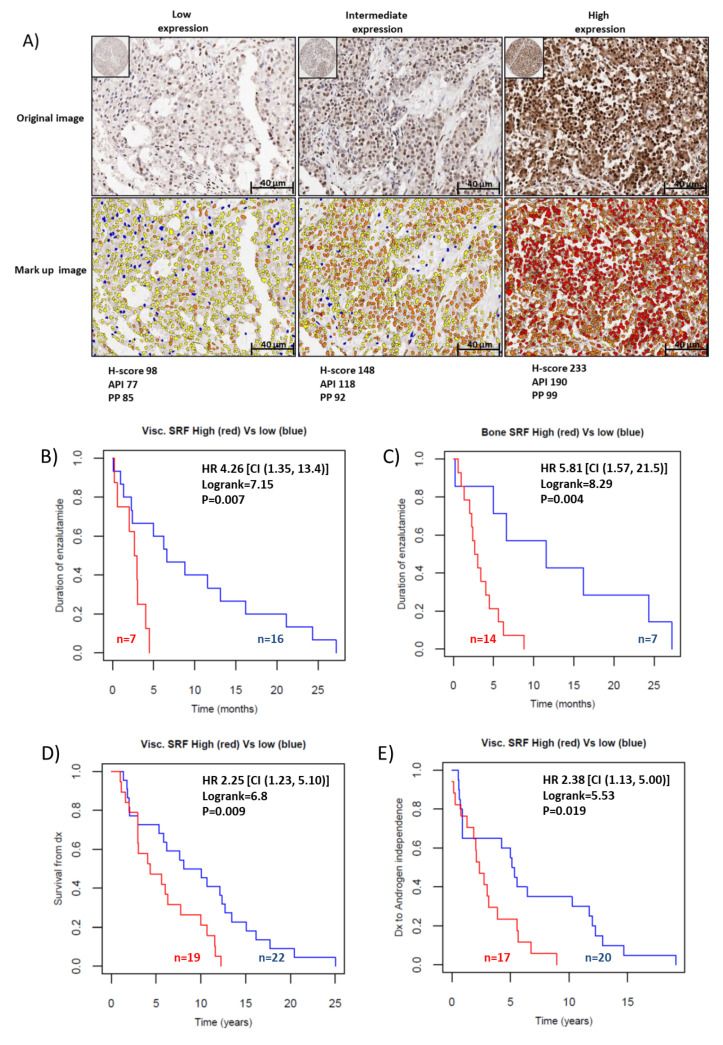
IHC staining for SRF was performed as described in the methods section. (**A**) Representative images of SRF staining with low, intermediate and high SRF nuclear protein expression. Top panel shows original images (20× magnification); bottom panel shows the markup images resulting from automatic quantification, using Visopharm’s digital analysis algorithms. (**B**,**C**) Kaplan–Meier cumulative survival from date of resistance to enzalutamide (indicated as duration to enzalutamide) stratified by patients with low and high average visceral (**B**) and bone (**C**) SRF nuclear expression (log-rank test, *p* = 0.007 and 0.004, respectively). (**D**) Kaplan–Meier cumulative survival from date of prostate cancer diagnosis stratified by patients with low and high average visceral SRF nuclear expression (log-rank test, *p* = 0.009). (**E**): Kaplan–Meier cumulative survival from date of resistance to androgen deprivation therapy stratified by patients with low and high average visceral SRF nuclear expression (log-rank test, *p* = 0.019).

One mechanism of action of enzalutamide is to inhibit AR translocation to the nucleus by binding AR ligand binding domain, competing with DHT and inhibiting AR dimerisation, necessary for nuclear translocation [15]. Considering that our results showed a significant decrease in AR nuclear localisation following inhibition of SRF with CCG1423 (Figure 3), we hypothesised that SRF expression could be associated with the response to enzalutamide in patients. Using IHC, we assessed SRF immunoreactivity in metastatic tissues from CRPC patients (Figure 5A–E). Of the 43 patients considered, 23 had received enzalutamide. There was no difference in SRF nuclear expression between patients who received enzalutamide and patients who did not receive it. Kaplan–Meier curves for the 23 patients who were treated with enzalutamide showed that high SRF expression levels were associated with shorter survival time on enzalutamide, for both bone and visceral metastases (Figure 5B,C). These data indicate that SRF expression is associated with the enzalutamide response, with higher levels of SRF protein expression associated with resistance to treatment. Kaplan–Meier analysis in this cohort of patients also validated our previous study [12], showing that SRF expression is associated with survival from diagnosis with prostate cancer (Figure 5D) and with time to developing resistance to androgen deprivation therapy (Figure 5E).

## 3. Discussion

Despite the emergence of next-generation anti androgens such as enzalutamide and abiraterone acetate, advanced prostate cancer is still challenging to treat due to development of CRPC and subsequent resistance to other available treatments, such as docetaxel. AR mutations and splice variants are among the main mechanisms of resistance to enzalutamide [2]. In an effort to overcome this resistance, novel approaches need to be investigated that focus on alternative ways of disrupting AR signalling without targeting the AR protein directly, which triggers AR resistant phenotypes. One such approach focuses on targeting AR co-factors and co-regulators [5]. In this study, we propose to inhibit SRF, a co-regulator of AR in prostate cancer [9,11], as a possible way to overcome resistance to enzalutamide.

The small molecule inhibitor CCG1423 [18] was used to inhibit SRF transcriptional activity in vitro and in vivo. Here, we showed that prostate cancer cells (both sensitive and resistant to hormone-therapy) respond to CCG1423 treatment, with LNCaP Abl-resistant cells being more responsive to CCG1423 than enzalutamide in terms of IC50 values. This result in itself suggests the possibility to offer additional treatments to patients who are resistant to hormone-therapy in general and enzalutamide in particular. Moreover, the combination of CCG1423 and enzalutamide resulted in increased response to enzalutamide, as indicated by the decrease in enzalutamide IC50 values in both cell lines. These data were confirmed in vivo in the LuCaP35 CR PDX line, where a lower TV was demonstrated following CCG1423 treatment, singly or in combination with enzalutamide. The CCG1423 dose (0.15 mg/kg) used in this study, which was chosen based on a previous study on insulin-resistance in skeletal muscle [19], was significantly lower when compared for example to the enzalutamide dose (0.50 mg/kg), which may be beneficial for patients in terms of side effects. Nevertheless, even at this low dose, after three weeks of CCG1423 treatment, a significant decrease in PSA levels was achieved as well as a significant reduction in TV from week 4.5 of treatment. Moreover, Kaplan–Meier analysis showed that, in contrast with single treatments, combination treatment provides significant survival benefit compared with the control. The fact that single treatments had an effect on TV but did not show a survival advantage compared to control indicates that longer time may be needed to be able to see the biological effect of CCG1423 translated into better survival. While further in vivo studies, both in terms of dose and length of treatment, are needed for CCG1423 as a single agent, the survival data for the combination are in line with our hypothesis that SRF inhibition enhances response to enzalutamide.

Since PSA decrease is linked to a disruption in AR transcriptional activity, and a relationship between AR and SRF has been previously shown [9,10,11], we looked at AR expression in the tissues from the PDX model and in cell lines following CCG1423 treatment. The fact that SRF inhibition with CCG1423 results in decreased AR nuclear localisation strongly supports the rationale of targeting AR co-factors, in this case SRF, as an alternative way of disrupting AR signalling. In terms of the mechanisms behind this observation, a link between AR nuclear translocation and SRF inhibition can be found in the decrease in α-tubulin acetylation following treatment with CCG1423. Post-translational modifications of α-tubulin have been shown to be associated with prostate cancer progression, with loss of acetylated α-tubulin a common feature in AR-negative castrate-resistant cell lines, suggesting a link between AR and acetylation of α-tubulin in prostate cancer [20]. Elevated levels of α-tubulin acetylation have also been associated with metastatic potential in breast cancer [21]. Boggs and colleagues have shown a relationship between increased α-tubulin acetylation and the aggressive behaviours of basal-like breast cancers in two independent cohorts of patients [21]. Acetylation of α-tubulin has been associated with higher dynein binding and increased intracellular protein trafficking [22], including AR nuclear translocation [23]. Moreover, a physical interaction between the N-terminal domain of AR and α-tubulin has been shown in prostate cancer [24], linking docetaxel mechanism of action with inhibition of AR nuclear translocation through regulation of α-tubulin. In line with these studies, here we showed that SRF inhibition with CCG1423 results in a significant decrease in AR nuclear localisation and acetylation of α-tubulin. These results may be explained by the fact that SRF is a transcriptional activator of αTAT1, a protein responsible for adding acetyl groups to α-tubulin [17], hence the effect of CCG1423 in reducing acetylation of α-tubulin and AR intracellular localisation. However, while our WB data show robust decrease in acetylation of α-tubulin for the combination treatment for both LNCaP parental and Abl cell lines (with or without DHT stimulation), the results for CCG1423 single treatment are less clear. Moreover, treatment with CCG1423 (singly or in combination with enzalutamide) shows the opposite effect on α-tubulin acetylation in the AR-negative PC3 cell line. The fact that single CCG1423 treatment varies in prostate cancer cell lines with different AR levels (LNCaP parental, LNCaP Abl and PC3), as well as in the same cell line (LNCaP Abl), depending on DHT stimulation, indicates that AR involvement in this process is important. Further studies are needed to investigate the role of α-tubulin acetylation, androgens levels and AR expression, as a link between SRF inhibition and AR sub-cellular localisation in prostate cancer.

SRF expression in tumour tissues from prostate cancer patients has been extensively characterised by our group, showing that elevated nuclear SRF expression occurs early on during prostate cancer progression [13] and is associated with castrate resistance [7], shorter survival from diagnosis [12] and decreased response to docetaxel [14]. Here, we confirmed our previous studies on SRF association with survival from diagnosis with prostate cancer, and CRPC [12] in an independent cohort of patients who died with CRPC. Importantly, we showed that elevated levels of SRF nuclear expression are associated with shorter time on enzalutamide, which we used as a proxy for response to this drug, suggesting that upregulation of SRF may lead to resistance to enzalutamide. Our in vitro and in vivo data suggest that treatment with CCG1423 inhibits AR translocation to the nucleus with decrease in cell viability, tumour volume and PSA. Therefore, it is tempting to speculate that patients with CRPC who are resistant to enzalutamide and show high levels of SRF would benefit from CCG1423 treatment or other SRF inhibitors. In addition to these enzalutamide-resistant patients, this approach would also benefit those patients who have not developed enzalutamide resistance yet. For these patients, combination treatment with an SRF inhibitor could potentially prolong responsiveness to enzalutamide as a more effective therapy. The fact that SRF nuclear expression in prostate cancer tissues can be measured by IHC, an effective and cheap test routinely used for many cancer biomarkers [25], and that SRF up-regulation occurs early during prostate cancer progression [13], not only makes SRF an attractive therapeutic target but also a possible predictive biomarker and companion diagnostic for SRF inhibitors.

## 4. Materials and Methods

### 4.1. Cell Culture

The LNCaP parental cells (ATCC) were cultured in Advanced RPMI-1640 medium supplemented with 10% Fetal Bovine Serum (FBS), 100 µL/mL streptomycin, 100 U/mL penicillin and 1% Hepes. The LNCaP Abl subline is a model for castrate resistance, with a 30-fold increase in AR transcriptional activity compared with the LNCaP parental line. LNCaP Abl cells were generated from the parental cell line as described previously [8] and cultured in Advanced RPMI-1640 medium supplemented with 10% charcoal-stripped FBS, 100 µL/mL streptomycin, 100 U/mL penicillin and 1% Hepes. PC3 cells (ATCC) were cultured in RPMI-1640 medium supplemented with 10% FBS, 100 µL/mL streptomycin, 100 U/mL penicillin. All cell lines were maintained at 37 ºC in a humidified atmosphere of 5% CO_2_ in air.

### 4.2. 3-(4,5)-dimethylthiazol-2-yl-2,5-diphenyltetrazolium Bromide (MTT) Cell Viability Assay

Three thousand cells per well were seeded in 96-well plates. The following day the cells were treated either with vehicle (DMSO), increasing concentrations of CCG1423 (MedChem Express) alone (parental: 10, 12, 14, 16, 18, 20 μM; Abl: 2.5, 5, 10, 12, 15, 20 μM), increasing concentrations of enzalutamide (MedChem Express) alone (parental: 0.2, 2, 20, 30, 60, 80 μM; Abl: 5, 10, 20, 40, 60, 80 μM) or all the 36 possible combinations of CCG1423 and enzalutamide. Cells were treated for five days and cellular viability was measured by MTT assays as previously described [26]. IC50 values were calculated using GraphPad Prism program (GraphPad, San Diego, California).

### 4.3. PDX Study and Statistical Analysis

The in vivo PDX experiment was approved by the University of Washington Institutional Animal Care and Use Committee (IRB number is IRB# 2341). Fox Chase CB17 SCID castrated male mice were implanted with LuCaP 35CR, a model of enzalutamide-resistant CRPC [27]. Once tumour volumes exceeded 100 mm^3^, animals were randomised to receive either vehicle control (*n* = 11), enzalutamide (ENZA; 50 mg/kg p.o.; 5 days on, 2 days off; *n* = 11), CCG1423 (CCG; 0.15 mg/kg i.p. 5 days on, 2 days off; *n* = 11) or enzalutamide plus CCG1423 (dosing regimen as previously noted). Blood was collected from the retro-orbital sinus 3 weeks after the beginning of the treatment to determine serum PSA levels. Animals were sacrificed at 6 weeks post enrolment, when tumours exceeded 1000 mm^3^, or when they became otherwise compromised. Tumour volumes (TV) and body weights (BW) were measured twice weekly. Average PSA values and TV for each treatment group were compared using *t* test assuming equal variance. * *p* < 0.05, ** *p* < 0.01. Kaplan–Meier cumulative survival curves were generated using Graphpad, Prism program. Thirteen animals whose death was not related to tumour growth (found dead or had ulcerating tumours) were excluded from the survival analysis. Five-fold increase in TV from the enrolment was considered as “death”. Hazard ratios from Mantel–Haenszel models and *p*-values from the relevant log rank tests were calculated.

### 4.4. Immunohistochemical (IHC) and Immunocytochemical (ICC) Staining

For IHC staining, antigen retrieval of the deparaffinised tissue sections was performed using a PT Link module (DAKO) at 95–99 °C for 20 min in a citric acid buffer (0.01 M, pH 6.0); this step was not necessary for ICC staining. Staining was performed using a DAKO autostainer Link 48 according to the manufacturer instructions. SRF antibody (Novus Biological NBP1-71927) and AR antibody (Santa Cruz AR (441): sc-7305) were used at a concentration of 1:500.

### 4.5. Scoring of AR Protein Immunoreactivity and Statistical Analysis

Following IHC, slides were scanned using an Aperio ScanScope XT system (Aperio Technologies) at 20× magnification. AR immunoreactivity was analysed by automatic quantification using Visopharm’s Oncotopix (Hoersholm, Denmark); the classification method used “Cell Classification” for nuclear and cytoplasmic assessment. AR expression was reported as H-Score [28]. Average AR H-Score values for each treatment group were compared using one-way ANOVA test for cytoplasmic AR staining (equal variance) and Welch’s ANOVA followed by post hoc Games–Howell test for nuclear AR staining (unequal variance). ** *p* < 0.01, * *p* < 0.05.

### 4.6. Western Blotting

Western blots were carried out as previously published [9]. The following primary antibodies were used: anti-AR (1:500, Santa Cruz), anti-α-tubulin (1:2000, Abcam), anti-acetylated α-tubulin (1:1000, abcam) and GAPDH (1:5000, Millipore). Enhanced Chemiluminescence (ThermoSCIENTIFIC, Waltham, MA, USA) was used to visualise protein bands on the membrane. Images of the membranes were taken using an Amersham Imager 600, and densitometric analysis was performed using ImageJ software.

### 4.7. Sample Collection/Tissue Microarray Description

Human tissue microarrays were constructed consisting of soft tissue metastases and bone metastases from 45 patients with advanced prostate cancer. Samples were obtained from patients who died of metastatic CRPC and who signed written informed consent for a rapid autopsy, under the aegis of the Prostate Cancer Donor Program at the University of Washington [29]. Two replicate 1 mm cores of soft tissue metastases and bone metastases were taken from every case where available, as described for a previous tissue microarray build from a different patient cohort [30]. The tissue microarrays were assembled using the Beecher Instruments Tissue-ArrayerTM (Beecher Instruments, Silver Spring, MD, USA). Of the 45 patients, 4 were excluded due to loss of tissue during staining. Out of these 41 patients, 23 had received enzalutamide treatment.

### 4.8. Scoring of SRF Protein Expression and Statistical Analysis

Cores with necrosis were excluded from our analysis. Nuclear immunoreactivity for SRF in soft tissue metastases and bone metastases was assessed by automatic quantification, using Visopharm’s Oncotopix (Hoersholm, Denmark); the classification method used “Threshold”. SRF expression was reported as H-Score. For the purpose of statistical analysis, the nuclear scores of SRF was divided into low and high expression. The cut-off values for low/high expression were calculated by dividing the patient H-Score data into quantiles, 1–99 in turn, and establishing the largest separation using the log-rank test. Kaplan–Meier cumulative survival curves were generated by dividing the data in two at the appropriate cut-off point, and assessing the times to the relevant endpoints, using the survival package (version 2.41–3) [31] in R (version 3.4.3) [32]. Hazard ratios from CoxPH models and *p*-values from the relevant log rank tests are also displayed.

## 5. Conclusions

Our study showed an alternative way to interfere with the AR signalling pathway, through inhibition of SRF. Considering that SRF expression has been associated with prostate cancer progression and resistance to enzalutamide in patients, we propose to target SRF as a therapeutic strategy to treat CRPC.

## Supplementary Materials

The following are available online at https://www.mdpi.com/2072-6694/12/12/3540/s1, Figure S1: Kaplan–Meier survival curves from date of enrolment for controls and single CCG1423/enzalutamide treatment groups.
