# Supplementary material for: Inhibition of Serum Response Factor Improves Response to Enzalutamide in Prostate Cancer
Source: Cancers (Basel). 2020 Nov 27;12(12):3540. doi: 10.3390/cancers12123540 (PMC7760758; doi:10.3390/cancers12123540)

cancers

Supplementary materials: Inhibition of Serum Response Factor Improves Response to Enzalutamide in Prostate Cancer

R. William Watson, Haleema Azam, Claudia Aura, Niamh Russell, Janet McCormack, Eva Corey, Colm Morrissey, John Crown, William M Gallagher and Maria Prencipe

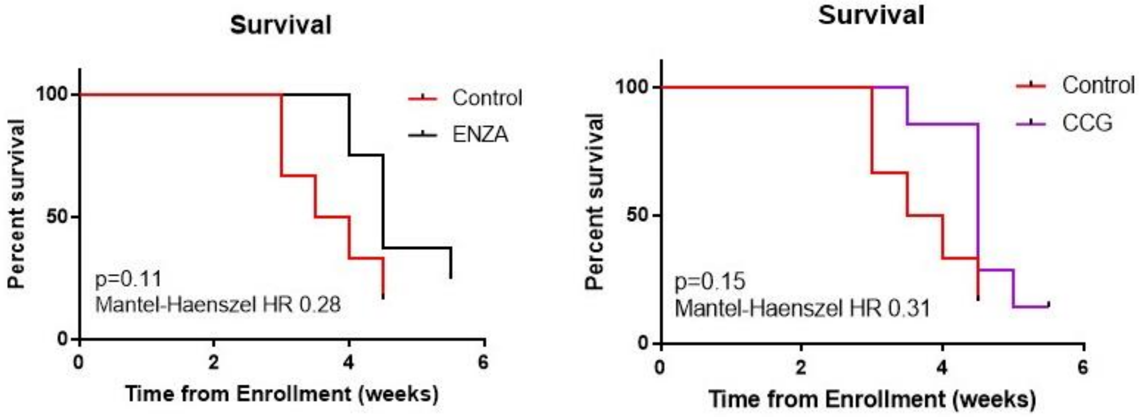

Figure S1. Kaplan–Meier survival curves from date of enrolment for controls and single CCG1423/enzalutamide treatment groups.

Original images

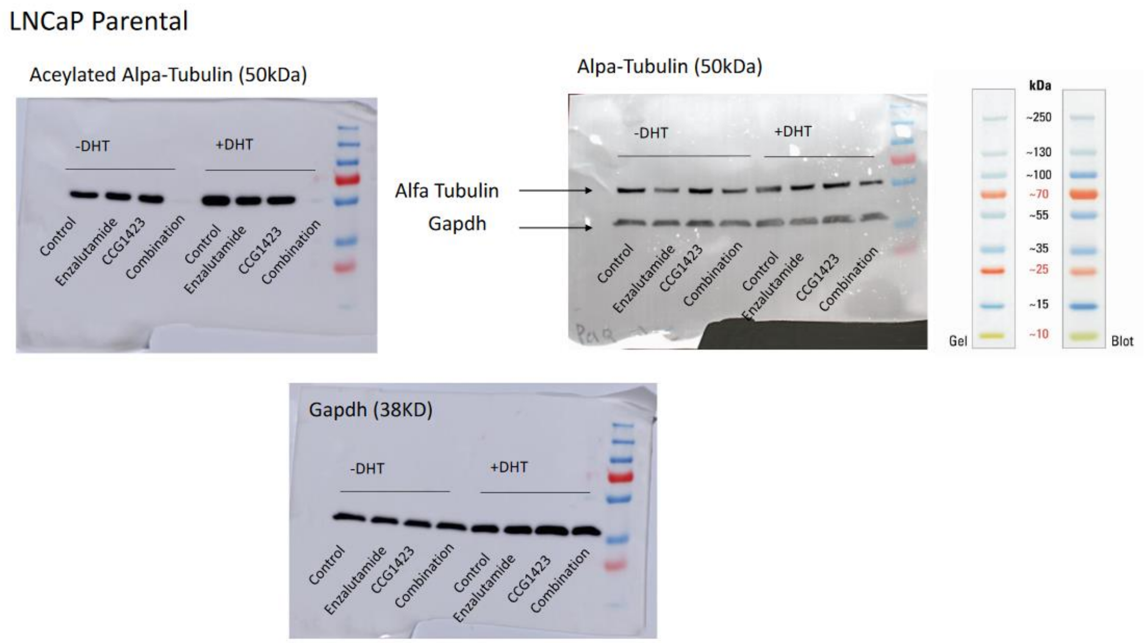

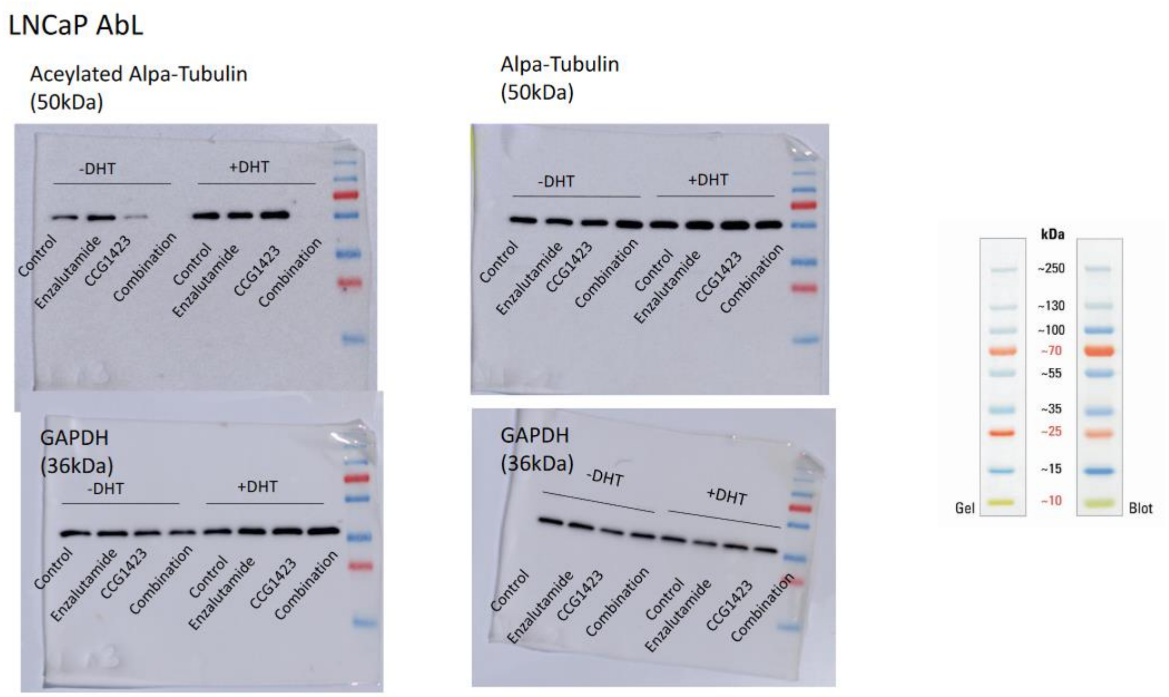

Figure 4. Protein expression of acetylated α-tubulin assessed by WB in LNCaP parental and Abl cells post-treatments

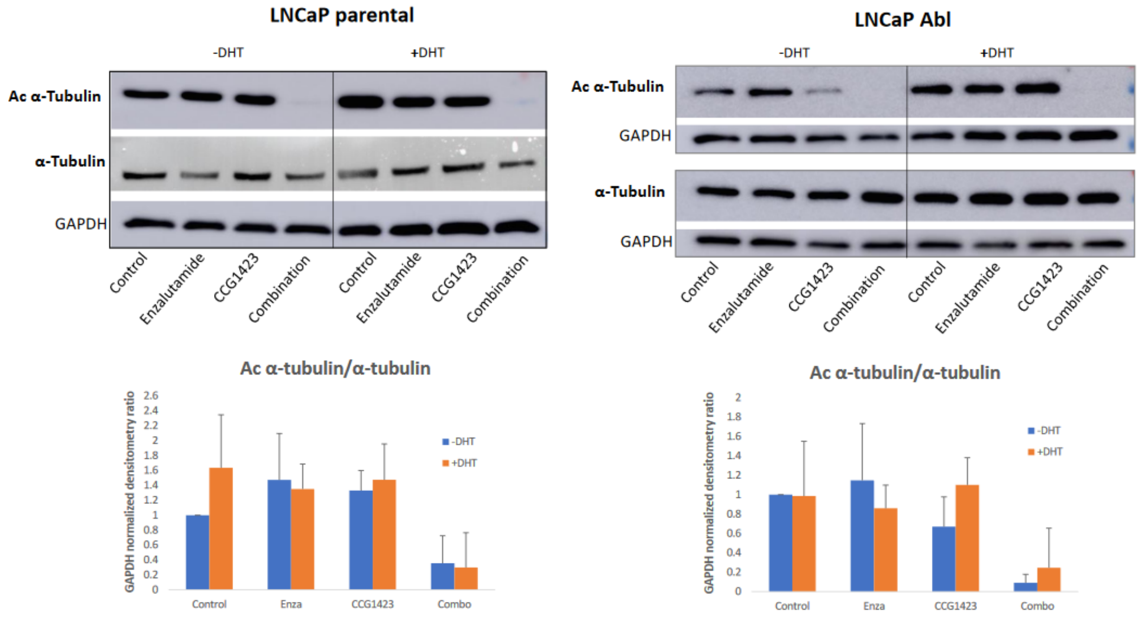

Supplement: Supplementary file 1 [file cancers-12-03540-s001.pdf]
